# Qual Deve Ser o Tratamento de Primeira Linha para o Fechamento de Persistência de Canal Arterial Hemodinamicamente Significativo em Bebês Prematuros?

**DOI:** 10.36660/abc.20201361

**Published:** 2022-03-10

**Authors:** Ufuk Cakir, Cuneyt Tayman

**Affiliations:** 1 Departamento de Pediatria Health Sciences University Ankara Bilkent City Hospital Ankara Turquia Divisão de Neonatologia, Departamento de Pediatria, Health Sciences University, Ankara Bilkent City Hospital, Ankara - Turquia

**Keywords:** Recém-Nascido Prematuro, Permeabilidade do Canal Arterial/cirurgia, Recém-Nascido de Baixo Peso, Ibuprofeno/uso terapêutico, Acetaminofen/uso terapêutico

## Abstract

**Fundamento:**

É importante saber qual medicamento usar como tratamento de primeira linha para fechar o duto.

**Objetivos:**

O objetivo deste estudo é comparar a eficácia e os efeitos colaterais das formas intravenosas (IV) de ibuprofeno e paracetamol e contribuir para a literatura investigando o primeiro medicamento selecionado no tratamento clínico da persistência do canal arterial (PCA).

**Métodos:**

Nosso estudo foi realizado entre janeiro de 2017 e dezembro de 2019. Foram incluídos no estudo bebês prematuros com peso ao nascer (PN) ≤1500 g e idade gestacional (IG) ≤32 semanas. No período do estudo, todos os bebês com persistência do canal arterial hemodinamicamente significativa (hsPCA) receberam ibuprofeno intravenoso (IV) como resgate como tratamento clínico primário ou tratamento com paracetamol IV se houvesse contraindicações para o ibuprofeno. Os pacientes foram divididos em dois grupos: pacientes que receberam ibuprofeno IV e pacientes que receberam paracetamol IV.

**Resultados:**

Desses pacientes, 101 receberam paracetamol IV e 169 receberam ibuprofeno IV. A taxa de sucesso do fechamento da PCA com o primeiro curso do tratamento foi de 74,3% no grupo de paracetamol IV e 72,8% no grupo de ibuprofeno IV (p=0,212).

**Conclusões:**

Nossos resultados mostram que o paracetamol IV é tão eficaz quanto o ibuprofeno IV no tratamento de primeira linha de hsPCA, podendo se tornar o tratamento preferencial para o controle de hsPCA.

## Introdução

A persistência do canal arterial hemodinamicamente significativo (hsPCA) é uma causa comum de morbimortalidade que afeta mais de 40% dos bebês prematuros.^1^ A hsPCA prolongado interrompe a hemodinâmica sistêmica, causando consequências clínicas negativas, como síndrome do desconforto respiratório (SDR), hemorragia pulmonar, displasia broncopulmonar (DBP), diminuição da oxigenação cerebral, transtorno do neurodesenvolvimento, hemorragia intraventricular (HIV), insuficiência renal aguda, intolerância nutricional, enterocolite necrosante (ECN), retinopatia da prematuridade (RP), sepse e tempo prolongado de internação hospitalar. Portanto, a PCA precisa ser tratada. Se houver sinais clínicos de PCA, ela deve ser tratada.^1 , 2^ Apesar de estar associada a todos esses desfechos negativos, tem-se questionado uma relação causal, uma vez que alguns estudos não mostraram redução da maioria dessas comorbidades ou suas consequências com o tratamento do canal arterial. Vale ressaltar que esses estudos não foram elaborados para definir o papel do canal arterial (CA) na predição de desfechos clínicos adversos, mas ainda existem muitas controvérsias a respeito do tratamento (farmacológico, cirúrgico, percutâneo), do momento e dos subgrupos específicos de bebês prematuros que se beneficiariam com o fechamento do CA.^3 - 7^

Os medicamentos mais utilizados para o fechamento farmacológico são os inibidores da ciclooxigenase (COX), principalmente a indometacina e o ibuprofeno, que bloqueiam a conversão do ácido araquidônico em prostaglandinas (PG). O sucesso relatado com ibuprofeno no tratamento da hsPCA é de 70–85%.^2^ Foram relatados efeitos colaterais negativos da terapia com ibuprofeno e indometacina, como vasoconstrição periférica, sangramento gastrointestinal e perfuração, diminuição da agregação plaquetária, hiperbilirrubinemia e insuficiência renal, embora sejam raros na prática clínica.^1^

O paracetamol, um inibidor da PG sintase, também pode ser usado no tratamento de hsPCA quando os inibidores da COX são contraindicados ou ineficazes e apresentam possíveis efeitos colaterais.^2^ O paracetamol se tornou uma alternativa cada vez mais comum ao ibuprofeno, havendo registros de estudos bem-sucedidos sobre paracetamol.^8^ O tratamento farmacológico da hsPCA continua sendo um desafio. Reduzir o surgimento de efeitos adversos e a necessidade de ligadura cirúrgica nesta área fortaleceu o propósito de identificar outros medicamentos adequados que sejam mais seguros e eficazes do que o ibuprofeno para bebês prematuros.^9^ Em estudos anteriores, que compararam a eficácia da terapia com paracetamol e ibuprofeno para hsPCA, foram utilizadas formas orais.^1 , 2 , 10 - 15^ Com base nesses estudos, uma metanálise recentemente publicada na Cochrane afirma que são necessários estudos antes de qualquer sugestão de uso rotineiro do paracetamol no tratamento da PCA em recém-nascidos. Combinando-se os resultados dos estudos incluídos, a taxa de sucesso do paracetamol para fechar uma PCA foi maior do que a do placebo e semelhante à do ibuprofeno e indometacina.^16^

O paracetamol parece ter sucesso para a hsPCA devido a possivelmente menos efeitos colaterais como uma opção de recuperação quando os inibidores de COX falham. No entanto, no tratamento de primeira linha da hsPCA, faltam informações sobre a taxa de sucesso do paracetamol em comparação com o ibuprofeno. Portanto, neste estudo, nosso objetivo foi comparar a eficácia e a segurança do ibuprofeno IV e do paracetamol IV para o fechamento farmacológico de PCA em bebês prematuros.

## Métodos

### Desenho do Estudo

Este estudo foi realizado entre janeiro de 2017 e dezembro de 2019 na unidade de terapia intensiva neonatal (UTIN) do Ankara Bilkent City Hospital. Este estudo apresenta desenho retrospectivo. Antes do estudo, obteve-se aprovação do comitê de ética local. Foram inscritos bebês prematuros com idade gestacional (IG) ≤32 semanas, peso ao nascer (PN) ≤1500 g, idade pós-natal ≥48 horas e com diagnóstico de hsPCA. Bebês prematuros com anomalia congênita importante, cardiopatia congênita, cardiopatia congênita dependente de ducto, que vieram a óbito nas primeiras 48 horas após o nascimento, foram excluídos do estudo.

### Características Clínicas e Demográficas

Variáveis perinatais incluindo IG, PN, sexo, escores de Apgar (1º e 5º minutos), administração de esteroide pré-natal, tratamento com paracetamol de 2 e 3 cursos, ligadura de PCA, hemorragia gastrointestinal, hemorragia pulmonar, SDR, HIV (grau ≥3), ECN (grau ≥2), DBP moderada ou grave, RP exigindo terapia a laser, sepse neonatal precoce (SNP), sepse neonatal tardia (SNT), duração de ventilação não invasiva (VNI), ventilação mecânica (VM) e suplementação de oxigênio (O_2_), dia de terapia nutricional enteral completa, tempo de internação hospitalar e mortalidade foram registrados para todos os bebês.

Definiu-se SNP como ≤72 horas e SNT >após 72 horas em prematuros hospitalizados na UTIN.^17^ Diagnosticou-se SDR como necessidade de administração de surfactante.^18^ Investigou-se HIV por ultrassonografia craniana realizada durante os primeiros 7 dias de vida (hemorragia intraparenquimatosa + HIV, HIV importante).^19^ Os critérios de Bell foram usados para o diagnóstico e estadiamento de ECN.^20^ Os bebês que receberam ≥30% de oxigênio com/sem qualquer pressão positiva com 36 semanas de idade pós-menstrual foram diagnosticados como DBP moderada ou grave.^21^ A RP foi analisada por oftalmologistas especializados com base na classificação internacional revisitada.^22^

### Avaliação Laboratorial e Radiológica

Antes e 24 horas após o primeiro curso do tratamento clínico, todos os pacientes foram submetidos a exames de função renal e hepática, incluindo creatinina sérica e nitrogênio ureico no sangue (NUS), aspartato amino transferase (AST) e alanina amino transferase (ALT), assim como estudos de imagem envolvendo ultrassonografia craniana e ecocardiografia (ECO).

### Persistência do Canal Arterial Hemodinamicamente Significativo

Realizou-se ECO em todos os pacientes na 72ª hora pós-natal. Determinou-se o diagnóstico de hsPCA de acordo com critérios clínicos e ecocardiográficos ( Tabela 1 ).^1 , 23 , 24^ O exame de ECO foi realizado por um cardiologista pediátrico. O eco-Doppler foi realizado por transdutor GE Vivid 7 Pro, 10S (GE Healthcare, Salt Lake City, Utah). A hsPCA foi inicialmente tratada com paracetamol IV ou ibuprofeno IV. Realizou-se ligadura cirúrgica em casos de persistência de hsPCA (após 3 cursos de tratamento com paracetamol ou ibuprofeno). O grupo não HsPCA foi selecionado de acordo com os mesmos critérios de exclusão, sendo composto por bebês sem hsPCA.

**Tabela 1:** – Persistência do canal arterial hemodinamicamente significativo

Características clínicas	Sopro
	Precórdio hiperdinâmico
	Pulsos pré-ductais delimitadores
	Piora do estado respiratório
	Pressão de pulso ampla
	Hipotensão
	Acidose metabólica
**Características ecocardiográficas**	Razão de aumento atrial esquerdo para a raiz da aorta
	Cardiomegalia
	Shunt da esquerda para a direita
	Canal aberto (>1,5 mm)
	Reversão do fluxo nas grandes artérias pós-ductais

### Tratamento Intravenoso de Paracetamol e Ibuprofeno

Durante o período do estudo, o tratamento com paracetamol IV ou ibuprofeno IV foi administrado como tratamento farmacológico de resgate primário para todos os bebês com hsPCA. No caso de contraindicação de ibuprofeno, iniciava-se o paracetamol. As contraindicações para o tratamento com ibuprofeno foram HIV ativa, trombocitopenia ou outros distúrbios de coagulação conhecidos, sepse importante, NEC suspeita ou confirmada, intolerância alimentar, perfuração intestinal, comprometimento significativo da função renal e hiperbilirrubinemia importante.^14 , 15 , 25^ Os pacientes foram divididos em dois grupos: pacientes que receberam paracetamol IV e pacientes que receberam ibuprofeno IV. Cada paciente elegível recebeu paracetamol IV (Parol, Atabay Ilac Kimya San., Istambul, Turquia) em uma dose de 15 mg/kg a cada 6 horas por 5 dias ou ibuprofeno IV (Intrafen; Gen Ilac, Ancara, Turquia) em uma primeira dose de 10 mg/kg seguida de 5 mg/kg em 24 e 48 horas por 3 dias.

### Seguimento dos Pacientes

Um dia após o tratamento, realizou-se avaliação ecocardiográfica por cardiologista pediátrico. Os pacientes com shunt ductal mínimo foram acompanhados regularmente por um neonatologista e um cardiologista pediátrico. Os pacientes que conseguiram o fechamento da PCA, mas apresentaram sinais e sintomas de reabertura posterior durante a hospitalização, foram reavaliados ecocardiograficamente e tratados de acordo com os resultados ecocardiográficos e com sua condição clínica.

A ingestão de líquidos foi iniciada em 70–80 mL/kg por dia e foi aumentada em incrementos de 10–20 mL/kg por dia, até um máximo de 150–160 mL/kg por dia para todos os pacientes inscritos no estudo. A hipotensão foi tratada com dopamina em pacientes nos quais o tratamento com líquidos falhou. Realizou-se ventilação de acordo com a gravidade do desconforto respiratório, utilizando pressão positiva contínua nasal ou VM. Pacientes com SNP ou SNT foram tratados de acordo com o protocolo da UTIN.

### Análise dos Dados

As análises estatísticas foram realizadas utilizando o software Statistical Package for Social Sciences (SPSS) versão 17 para Windows (SPSS Inc., Chicago, Illinois). A distribuição normal dos dados foi realizada pelo teste de Kolmogorov-Smirnov. O teste U de Mann-Whitney para variáveis contínuas não paramétricas em amostras independentes e os testes qui-quadrado ou exato de Fisher para variáveis categóricas foram usados para a comparação dos grupos. Os resultados foram apresentados como mediana (intervalo interquartil) para variáveis contínuas e percentuais e distribuição de frequência para variáveis categóricas. Definiu-se um valor de p bicaudal de 0,05 como ponto de corte para significância estatística.

## Resultados

**Figura 1:**
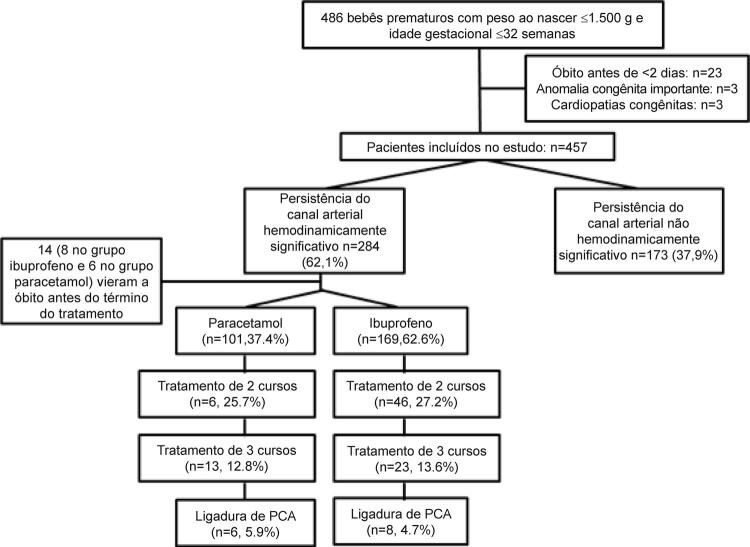
– Fluxograma da população estudada. PCA: persistência do canal arterial.

No total, 486 bebês prematuros com PN ≤1.500 g e IG ≤32 semanas foram admitidos à nossa UTIN durante o período do estudo. De acordo com os critérios de exclusão, 29 bebês prematuros foram excluídos do estudo. Dos 457 bebês prematuros restantes, 284 bebês foram diagnosticados com hsPCA. 14 bebês vieram a óbito antes do início da terapia medicamentosa. Os 270 pacientes restantes com hsPCA, incluindo 101 pacientes recebendo paracetamol IV e 169 pacientes recebendo ibuprofeno IV, foram incluídos no estudo e analisados. A IG e o PN medianos de todos os pacientes elegíveis foram 27,7 (2,2) semanas e 1006 (324) g (mediana [intervalo interquartil]), respectivamente. A taxa de hsPCA foi de 62,1% (284/457) entre os bebês prematuros. A taxa de sucesso do fechamento da PCA com o primeiro curso foi de 74,3% (75/101) no grupo de paracetamol IV e 72,8% (123/169) no grupo de ibuprofeno IV (p=0,212). A taxa de sucesso do fechamento do PCA com o segundo curso foi de 50% (13/26) no grupo de paracetamol IV e 50% (23/46) no grupo de ibuprofeno IV. A necessidade de segundo curso do tratamento foi de 27,5% (26/101) no grupo paracetamol e 27,2% (46/169) no grupo ibuprofeno (p=0,312). A taxa de sucesso do fechamento de PCA com o terceiro curso foi de 53% (7/13) no grupo paracetamol IV e 65% (15/23) no grupo ibuprofeno IV. A necessidade de terceiro curso de tratamento foi de 12,8% (13/101) no grupo paracetamol e 13,6% (23-169) no grupo ibuprofeno (p=0,191). A taxa de ligadura foi de 5,9% no grupo paracetamol e 4,7% no grupo ibuprofeno ( Figura 1 ). Não houve diferença estatística entre os grupos (p=0,303). Os resultados foram semelhantes entre os grupos paracetamol e ibuprofeno em termos de características clínicas e demográficas, desfechos clínicos, testes de função hepática e renal ( Tabelas 2 , 3 e 4 ).

**Tabela 2:** – Características Clínicas e Demográficas da População do Estudo

Características clínicas e demográficas	Paracetamol (n: 101, 37,4%)	Ibuprofeno (n: 169, 62,6%)	Valor de p
Idade gestacional, semanas,^a^	28 (2,8)	28 (2)	0,653
Peso ao nascer, g,^a^	1042 (426)	1020 (290)	0,329
Masculino,^b^	53 (52,4)	79 (47,6)	0,381
Apgar 1 minuto,^a^	5 (2)	5 (2)	0,112
Apgar 5 minutos,^a^	7 (2)	8 (1)	0,153
Esteroide pré-natal,^b^	71 (70,2)	119 (68,6)	0,124
Duração da VM, dias,^a^	3 (8)	2 (5)	0,270
Duração da VNI, dias,^a^	12 (16)	9 (12)	0,980
Suplementação de oxigênio, dias,^a^	42 (36)	30 (33)	0,388
Dia de terapia nutricional enteral completa, dias,^a^	17 (11)	16 (8)	0,131
Tempo de hospitalização, dias,^a^	76 (45)	66 (30)	0,861

*VM: ventilação mecânica; VNI: ventilação não invasiva.^a^ Mediana (intervalo interquartil),^b^ n (%).*

**Tabela 3:** – Desfechos clínicos dos grupos de estudo

Características clínicas e demográficas	Paracetamol (n: 101, 37,4%)	Ibuprofeno (n: 169, 62,6%)	Valor de p
SDR,^a^	84 (83,1)	131 (77,5)	0,279
HIV, grau ≥3,^a^	12 (11,8)	13 (7,7)	0,279
ECN, estágio ≥2,^a^	3 (2)	4 (2,3)	0,524
DBP,^a^	22 (21,7)	34 (20,1)	0,421
RP,^a^	15 (14,8)	24 (14,2)	0,255
SNP,^a^	18 (17,8)	28 (16,5)	0,867
SNT,^a^	35 (34,6)	44 (26)	0,454
Sangramento gastrointestinal,^a^	-	4 (2,3)	0,321
Hemorragia pulmonar,^a^	2 (2)	5 (2,9)	0,514
Mortalidade,^a^	17 (19,8)	19 (11,2)	0,131
2^o^ curso do tratamento,^a^	26 (25,7)	46 (27,2)	0,312
3^o^ curso do tratamento,^a^	13 (12,8)	23 (13,6)	0,191
Ligadura do PCA,^a^	6 (5,9)	8 (4,7)	0,303

*^a^n (%). DBP: displasia broncopulmonar; SNP: sepse neonatal precoce; PCA: persistência do canal arterial; HIV: hemorragia intraventricular; SNT: sepse neonatal tardia; ECN: enterocolite necrosante; SDR: síndrome do desconforto respiratório; RP: retinopatia da prematuridade.*

**Tabela 4:** – Avaliação dos testes de função hepática e renal após o primeiro curso de tratamento

Exames laboratoriais	Paracetamol intravenoso		Valor de p	Ibuprofeno intravenoso		Valor de p	Valor de p
	
	Pré-tratamento	Pós-tratamento		Pré-tratamento	Pós-tratamento		
NUS, (mg/dL),^a^	40 (24)	44 (28)	0,352	42 (25)	42 (11-92)	0,926	0,667
Creatinina sérica (mg/dL),^a^	0,8 (0,5)	0,8 (0,3)	0,339	0,7 (0,4)	0,8 (0,4)	0,116	0,452
AST sérica (Unidades/L),^a^	28 (10)	28 (17)	0,758	28 (13)	28 (15)	0,995	0,844
ALT sérica (unidades/L),^a^	23 (12)	20 (13)	0,571	24 (14)	22 (12)	0,237	0,707

ALT: alanina aminotransferase; AST: aspartato amino transferase; NUS: nitrogênio ureico no sangue. *valor de p para medidas pós-tratamento entre os grupos. aMediana (intervalo interquartil).

## Discussão

Nossos resultados mostraram que o paracetamol IV e o ibuprofeno IV são igualmente eficazes quando usados como a opção de tratamento de primeira linha para o fechamento de PCA. Além disso, ambos os medicamentos intravenosos foram bem tolerados em termos de efeitos colaterais renais e hepáticos, e complicações gastrointestinais e pulmonares. Além disso, não houve diferença entre os grupos em relação à morbimortalidade prematura. Como a maioria (90%) dos casos de HIV e hemorragia pulmonar ocorre antes das 72 horas de vida, qualquer tratamento iniciado além desse período não deve ser capaz de reduzir sua incidência, portanto, não seria de se esperar nenhuma diferença entre os medicamentos usados em nosso estudo.^26^

O canal arterial é uma formação anatômica vital que conecta a circulação pulmonar e sistêmica no feto. Os principais fatores que causam a permeabilidade do CA na vida intrauterina são a baixa pressão de oxigênio, PG e óxido nítrico. O aumento dos níveis de oxigênio e a diminuição da prostaglandina E2 (PGE2) imediatamente após o parto permitem o fechamento funcional do canal. A persistência do canal interrompe a hemodinâmica em bebês prematuros e contribui para a morbimortalidade relacionada à prematuridade.^27^ Portanto, quando a hsPCA é detectada, dois fatores principais (níveis de oxigênio e PG) que propiciam a vasodilatação do canal devem ser manipulados para garantir o fechamento do canal. Indometacina, ibuprofeno e paracetamol, que inibem a síntese de PG a partir do ácido araquidônico, proporcionando vasoconstrição, são usados para o fechamento do canal. A PG sintase é a principal enzima que converte o ácido araquidônico em PG. Essa enzima possui duas atividades catalíticas, incluindo COX (-1, -2, -3) e peroxidase. A indometacina e o ibuprofeno inibem as enzimas COX-1 e -2, e o paracetamol inibe a enzima COX-3 e a peroxidase, inibindo assim a síntese de PG. Enquanto a peroxidase, a enzima-alvo do paracetamol, pode ser ativada em níveis baixos de peróxido, a COX, a enzima-alvo do ibuprofeno, é ativada em níveis mais altos de peróxido. Portanto, o paracetamol é mais eficaz do que o ibuprofeno na hipóxia.^2^

Considerando os efeitos colaterais da indometacina e do ibuprofeno, novos métodos de tratamento foram necessários para reduzir a necessidade de ligadura. Considerou-se o paracetamol oral eficaz no tratamento de PCA em 5 pacientes com PCA que não responderam ao ibuprofeno na primeira série de casos relatada por Hammerman et al.,^28^ Nos primeiros estudos, o paracetamol não foi usado como medicamento de primeira linha, mas como alternativa aos casos em que os inibidores da COX eram ineficazes ou contraindicados.^28^ Em seguida, o paracetamol foi usado como tratamento de primeira linha para o fechamento do canal arterial.^1 , 2 , 10^ Estudos anteriores foram realizados para investigar a eficácia e a segurança do ibuprofeno oral em comparação com o paracetamol oral para esclarecer se o paracetamol pode ser usado como tratamento de primeira linha para o fechamento do canal em bebês prematuros. De forma geral, em estudos anteriores, a eficácia e a confiabilidade das formas orais de medicamentos usados no tratamento de hsPCA foram avaliadas.^1 , 29 - 34^

Nosso estudo, além de determinar a eficácia do paracetamol IV no tratamento da hsPCA, teve como objetivo compará-lo com o ibuprofeno IV. Com base em nossos achados, ao passo que as taxas de fechamento de hsPCA foram de 74,3% com paracetamol IV, essa proporção foi de 72,8% com ibuprofeno IV, e não houve diferença entre os grupos. Em muitos estudos, essa taxa ficou em aproximadamente 70–85%, semelhante aos resultados de nosso estudo. Resultados semelhantes foram encontrados em nosso estudo para efeitos colaterais, complicações e desfechos clínicos.^2^ No entanto, verificou-se que o número de casos nos grupos de estudos anteriores variou de 10 a 80.^1 , 2 , 10 , 11 , 35^ Portanto, nossos resultados podem ser mais robustos ou mais fortes e confiáveis do que outros estudos devido ao grande número de casos.

Embora haja evidências que mostram que o paracetamol é tão eficaz quanto o ibuprofeno, há estudos que encontraram resultados conflitantes. Por exemplo, Lu et al.^12^ mostraram que o paracetamol foi menos eficaz do que o ibuprofeno para o fechamento de PCA em recém-nascidos, e esse efeito foi reduzido ainda mais em bebês de muito baixo peso ao nascer (MBPN) ou extremamente baixo peso ao nascer.^12^ Da mesma forma, em um estudo de Sallmon, relatou-se que em paralelo à taxa de fechamento de PCA de 27,5% observada após o tratamento com paracetamol, a taxa de fechamento total de PCA foi de apenas 21,1% após a administração de paracetamol em bebês com MBPB.^36^ Além disso, alguns estudos relataram que o paracetamol não é tão eficaz quanto o ibuprofeno.^37 , 38^ Geralmente, o paracetamol é utilizado como opção alternativa em alguns pacientes nos quais o ibuprofeno não poderia ser utilizado, como bebês prematuros com sepse, com estado geral ruim, cujos órgãos apresentam funções inadequadas. Logicamente, o possível sucesso do paracetamol é diminuído nesses pacientes.^15^ Portanto, como no nosso estudo, seria mais apropriado avaliar que o paracetamol possa ser usado como primeira escolha com base nos resultados de estudos que compararam a eficácia do paracetamol e do ibuprofeno no tratamento de hsPCA. Nosso estudo mostrou que o paracetamol IV foi similarmente eficaz ao ibuprofeno IV no tratamento de HsPCA. Portanto, sugerimos que o paracetamol IV possa ser usado como o tratamento de primeira linha para hsPCA, bem como o ibuprofeno IV.

Estudos anteriores compararam a eficácia e a segurança das formas orais dos medicamentos para o tratamento de hsPCA.^1 , 2 , 10 - 13^ Além disso, existem poucos estudos sobre as formas IV. Um estudo de Roofthooft et al.^14^ relatou que o tratamento com paracetamol IV não é eficaz para fechamento de PCA em bebês com MBPN após falha do tratamento com ibuprofeno IV.^14^ Neste estudo, a terapia com paracetamol não foi recomendada para fechamento de PCA em bebês >2 semanas de idade após o nascimento. No entanto, relatou-se a eficácia do paracetamol quando usado como tratamento de primeira linha para PCA. Além disso, Valerio et al.^25^ apontaram que o paracetamol IV é eficaz no fechamento de PCA tanto para terapia de “atenção primária” quanto de “recuperação”.^25^ Em outro estudo que comparou o paracetamol IV e o ibuprofeno IV, afirmou-se que o paracetamol poderia se tornar o tratamento preferencial para o tratamento de PCA, principalmente devido ao seu perfil mais favorável em termos de efeitos colaterais.^9^ Nossos resultados também corroboram essas informações. Além disso, os efeitos colaterais do ibuprofeno às vezes são uma desvantagem para sua recomendação.^10 , 12^ Em alguns estudos, semelhantes aos nossos resultados, relata-se que não há diferença em termos do perfil de efeitos colaterais do paracetamol e do ibuprofeno.^1 , 2 , 9 , 11^ Além disso, corroborando os estudos anteriores, verificamos que as taxas de ligadura cirúrgica diminuíram de forma semelhante em bebês com hsPCA tratados com paracetamol ou ibuprofeno.^1 , 2 , 11^

No entanto, ainda está sendo investigado qual medicamento será administrado da maneira mais segura e eficaz. Apesar de todos esses resultados contraditórios, uma recente metanálise publicada na Cochrane por Ohlsson e Shah^16^ afirmou que são necessários mais estudos antes do uso rotineiro de paracetamol para o tratamento de primeira linha de hsPCA.^16^ Acreditamos que os resultados de nosso estudo lançarão luz sobre essa questão. De acordo com nossos resultados, quando o paracetamol IV foi administrado como a opção de tratamento de primeira linha para o fechamento de PCA, ele foi considerado tão eficaz quanto o ibuprofeno IV sem quaisquer efeitos colaterais.

Nosso estudo teve algumas limitações devido ao seu caráter retrospectivo. Outros parâmetros, como débito urinário por hora, nível de bilirrubina dos pacientes nos grupos de tratamento, não puderam ser avaliados. Faltam dados que recomendem o tratamento de primeira linha da PCA em termos de morbidades, pois há outros fatores que devem ser considerados. Por exemplo: o único medicamento que pôde reduzir a HIV nos estudos foi a indometacina precoce. Seria mais apropriado mostrar a eficácia do paracetamol no fechamento da PCA e sugerir estudos multicêntricos randomizados que pudessem atestar sua segurança e capacidade de redução de desfechos clínicos negativos.

## Conclusões

Quando o paracetamol foi usado como a opção de tratamento de primeira linha para o tratamento clínico de PCA, verificou-se que foi tão eficaz quanto o ibuprofeno. Além disso, não houve diferença entre os dois medicamentos em termos de morbimortalidade prematura. Estudos multicêntricos randomizados controlados sobre este assunto ajudarão a determinar o tratamento de primeira linha para hsPCA.
