# Supplementary material for: Qual Deve Ser o Tratamento de Primeira Linha para o Fechamento de Persistência de Canal Arterial Hemodinamicamente Significativo em Bebês Prematuros?
Source: Arq Bras Cardiol. 2022 Mar 10;118(3):548–55. [Article in Portuguese] doi: 10.36660/abc.20201361 (PMC8959034; doi:10.36660/abc.20201361)
Supplement: Translation

# Translation: What Should be the First-line Treatment for the Closure of Hemodynamically Significant Patent Ductus Arteriosus in Premature Infants?

**Authors:** Ufuk Cakir, Cuneyt Tayman

**Affiliations:** 1 Turkey Division of Neonatology, Department of Pediatrics, Health Sciences University, Ankara Bilkent City Hospital, Ankara - Turkey

**Keywords:** Infant, Premature, Ductus Arteriosus, Patent/surgery, Infant, Low Birth Weight, Ibuprofen/therapeutic use, Acetaminophen/therapeutic use

## Abstract

**Background:**

It is important which medicine to use as a first-line treatment to close the duct.

**Objectives:**

The aim of this study is to compare the effectiveness and side effects of intravenous (IV) forms of ibuprofen and paracetamol and to contribute to the literature investigating the first drug selected in the medical treatment of patent ductus arteriosus (PDA).

**Methods:**

Our study was conducted between January 2017 and December 2019. Premature infants with birth weight (BW) ≤1500 g and gestational age (GA) ≤32 weeks were included in the study. In the study period, all infants with hemodynamically significant patent ductus arteriosus (hsPDA) were given rescue intravenous (IV) ibuprofen as a primary medical treatment or IV paracetamol treatment if there were contraindications for ibuprofen. The patients were divided into two groups: patients receiving IV ibuprofen and patients receiving IV paracetamol.

**Results:**

Of these patients, 101 were given IV paracetamol and 169 were given IV ibuprofen. The success rate of PDA closure with first-course treatment was 74.3% in the IV paracetamol group and 72.8% in the IV ibuprofen group (p=0.212).

**Conclusions:**

Our results show that IV paracetamol is as effective as IV ibuprofen in the first-line treatment of hsPDA, and can become the preferred treatment for the management of hsPDA.

## Introduction

Hemodynamically significant patent ductus arteriosus (hsPDA) is a common cause of morbidity and mortality affecting more than 40% of premature babies.^1^ Prolonged hsPDA disrupts systemic hemodynamics causing negative clinical consequences such as respiratory distress syndrome (RDS), pulmonary hemorrhage, bronchopulmonary dysplasia (BPD), decrease in cerebral oxygenation, neurodevelopmental maturation disorder, intraventricular hemorrhage (IVH), acute renal failure, nutritional intolerance, necrotizing enterocolitis (NEC), retinopathy of prematurity (ROP), sepsis, and prolonged length of hospital stay. Therefore, it needs to be treated. If clinical signs of PDA exist, it should be treated.^1 , 2^ Despite being associated with all these negative outcomes, a causative relationship has been questioned since some studies did not show reduction of most of these comorbidities or its consequences with treatment of ductus arteriosus. It is noteworthy that these studies were not designed to define the role of the ductus arteriosus (DA) in the prediction of adverse clinical outcomes but there are still many controversies concerning the treatment (pharmacological, surgical, percutaneous), timing, and the specific subgroups of premature infants that would benefit from the closure of DA.^3 - 7^

The most commonly used drugs aiming at pharmacological closure are cyclooxygenase (COX) inhibitors, mainly indomethacin and ibuprofen, which block the conversion of arachidonic acid to prostaglandins (PG). The success reported with ibuprofen in the treatment of hsPDA is 70–85%.^2^ Negative side effects of ibuprofen and indomethacin therapy such as peripheral vasoconstriction, gastrointestinal bleeding and perforation, decreased platelet aggregation, hyperbilirubinemia and kidney failure have been reported, although they are rare in clinical practice.^1^

Paracetamol, a PG synthase inhibitor, can also be used in the treatment of hsPDA when COX inhibitors are contraindicated or ineffective and have potential side effects.^2^ Paracetamol has become an increasingly common alternative to ibuprofen, and studies on paracetamol have also been reported to be successful.^8^ The pharmacological treatment of hsPDA remains challenging. Reducing the emergence of adverse effects and the need for surgical ligation in this area has strengthened the purpose of identifying other suitable drugs that are safer and more effective than ibuprofen for premature babies.^9^ In previous studies comparing the effectiveness of paracetamol and ibuprofen therapy for hsPDA, oral forms have been used.^1 , 2 , 10 - 15^ Based on these studies, recent a Cochrane metanalysis states that studies are needed before any suggestions are made for routine use of paracetamol in the treatment of PDA in the newborns. When the results of the included studies were combined, the success rate for paracetamol to close a PDA was higher than that of placebo and similar to that of ibuprofen and indomethacin.^16^

Paracetamol seems to be successful for hsPDA due to possibly fewer side effects as a recovery option where COX inhibitors fail. However, in the first-line treatment of hsPDA, information on the success rate of paracetamol compared to ibuprofen is lacking. Therefore, in this study, we aimed to compare the efficacy and safety of IV ibuprofen and IV paracetamol for pharmacological closure of PDA in premature infants.

## Methods

### Study Design

This study was conducted between January 2017 and December 2019 in the neonatal intensive care unit (NICU) of Ankara Bilkent City Hospital. This study was designed retrospectively. Ethical committee approval was obtained from the local ethical committee prior to the study. Preterm infants with gestational age (GA) ≤32 weeks, birth weight (BW) ≤1500 g, postnatal age ≥48 hours, and diagnosed with hsPDA were enrolled. Preterm infants with major congenital anomaly, congenital heart disease, ductus dependent congenital heart disease, who died within the first 48 hours after birth, were excluded from the study.

### Demographic and Clinical Characteristics

Perinatal variables including GA, BW, gender, Apgar scores (1^st^and 5^th^minutes), antenatal steroids administration, 2- and 3-course paracetamol treatment, PDA ligation, gastrointestinal bleeding, pulmonary hemorrhage, RDS, IVH (grade ≥3), NEC (grade≥2), moderate or severe BPD, ROP requiring laser therapy, early-onset neonatal sepsis (EOS), late-onset sepsis (LOS), duration of non-invasive ventilation (NIV), mechanical ventilation (MV) and oxygen (O_2_) supplementation, day of full enteral feeding achievement, length of hospital stay and mortality were recorded for all infants.

EOS was defined as ≤72 hours and LOS >after 72 hours in preterm infants hospitalized in the NICU.^17^ RDS was diagnosed as requirement for surfactant administration.^18^ IVH was searched by cranial ultrasonography performed during the first 7 days of life (intraparenchymal hemorrhage + IVH, large IVH).^19^ Bell’s criteria were used for the diagnosis and staging of NEC.^20^ Infants receiving ≥30% oxygen with/without any positive pressure at postmenstrual age of 36 weeks were diagnosed as moderate or severe BPD.^21^ ROP was screened by specialized ophthalmologists based on the international classification revisited.^22^

### Laboratory and Radiological Evaluation

Before and 24 hours after the first course of medical treatment, all patients were evaluated for renal and liver function tests including serum creatinine and blood urea nitrogen (BUN), aspartate amino transferase (AST), and alanine amino transferase (ALT), as well as imaging studies involving cranial ultrasonography, and echocardiography (ECHO).

### Hemodynamically Significant Patent Ductus Arteriosus

ECHO was performed on all patients at postnatal 72^nd^hour. Diagnosis of hsPDA was determined according to clinical and ECHO criteria ( Table 1 ).^1 , 23 , 24^ ECHO examination was performed by a pediatric cardiologist. Doppler ECHO was performed using a GE Vivid 7 Pro, 10S transducer (GE Healthcare, Salt Lake City, Utah). hsPDA was initially treated with either IV paracetamol or IV ibuprofen. Surgical ligation was performed if hsPDA persisted (despite 3 courses of paracetamol or ibuprofen treatment). The non-hsDPA group was selected according to the same exclusion criteria, and consisted of infants without hsPDA.

**Table 1:** – Hemodynamically Significant Patent Ductus Arteriosus

Clinical characteristics	Murmur
	Hyperdynamic precordium
	Bounding preductal pulses
	Worsening respiratory status
	Wide pulse pressure
	Hypotension
	Metabolic acidosis
**Echocardiographic characteristics**	Increased left atrium to aorticroot ratio
	Cardiomegaly
	Left-to-right shunting
	Large open ductus (>1.5 mm)
	Reversal of flow in postductal major arteries

### Intravenous Treatment of Paracetamol and Ibuprofen

During the study period, IV paracetamol or IV ibuprofen treatment was given as a primary rescue pharmacological treatment to all infants with hsPDA. If ibuprofen was contraindicated, paracetamol was started. Contraindications for ibuprofen treatment were active IVH, thrombocytopenia or other known clotting disorders, severe sepsis, suspected or confirmed NEC, feeding intolerance, intestinal perforation, significant impairment of renal function, and severe hyperbilirubinemia.^14 , 15 , 25^ The patients were divided into two groups: patients receiving IV paracetamol and patients receiving IV ibuprofen. Each eligible patient received either IV paracetamol (Parol, Atabay Ilac Kimya San., Istanbul, Turkey) at a dose of 15 mg/kg every 6 hours for 5 days or IV ibuprofen (Intrafen; Gen Ilac, Ankara, Turkey) at an initial dose of 10 mg/kg followed by 5 mg/kg at 24 and 48 hours for 3 days.

### Patient Follow-up

One day after the treatment, an ECHO evaluation was performed by a pediatric cardiologist. Patients with minimal ductal shunting were followed up regularly by a neonatologist and a pediatric cardiologist. Patients who achieved PDA closure but had signs and symptoms of reopening later during hospitalization were re-evaluated by ECHO and were treated according to their ECHO findings and clinical condition.

Fluid intake was started at 70–80 mL/kg per day and was increased by increments of 10–20 mL/kg each day, to a maximum of 150–160 mL/kg per day for all patients enrolled in the study. Hypotension was treated with dopamine for patients in which fluid treatment had failed. Ventilation was supported according to the severity of respiratory distress, using nasal continuous positive airway pressure or MV. Patients with EOS or LOS were treated according to the NICU protocol.

### Data Analysis

Statistical analyses were performed using Statistical Package for Social Sciences (SPSS) version 17 for Windows (SPSS Inc, Chicago, Illinois). Normal distribution of data was performed by using Kolmogorov-Smirnov test. Mann-Whitney U-test for non-parametric continuous variables in independent samples and chi-square or Fisher’s exact tests for categorical variables were used for the comparison of the groups. The results were expressed as median (interquartile range) for continuous variables as well as percentage and distribution of frequency for categorical variables. Two-sided p-value of 0.05 was set as the cut-off for statistical significance.

## Results

A total of 486 preterm infants with BW ≤1500 g and GA ≤32 weeks were admitted to our NICU during the study period. According to the exclusion criteria, 29 preterm infants were excluded from the study. Of the remaining 457 preterm infants, 284 infants were diagnosed with hsPDA. 14 infants died before medical therapy was initiated. The remaining 270 patients with hsPDA, involving 101 patients receiving IV paracetamol and 169 patients receiving IV ibuprofen, were included in the study and analyzed. Median GA and BW of all eligible patients were 27.7 (2.2) weeks and 1006 (324) g (median [interquartile range]), respectively. The rate of hsPDA was 62.1% (284/457) among preterm infants. The success rate of PDA closure with the first course was 74.3% (75/101) in the IV paracetamol group and 72.8% (123/169) in the IV ibuprofen group (p=0.212). The success rate of PDA closure with the 2^nd^course was 50% (13/26) in the IV paracetamol group and 50% (23/46) in the IV ibuprofen group. The 2^nd^course treatment requirement was 27.5% (26/101) in the paracetamol group and 27.2% (46/169) in the ibuprofen group (p=0.312). The success rate of PDA closure with the 3^rd^course was 53% (7/13) in the IV paracetamol group and 65% (15/23) in the IV ibuprofen group. Third course treatment requirement was 12.8% (13/101) in the paracetamol group and 13.6% (23-169) in the ibuprofen group (p=0.191). The ligation rate was 5.9% in the paracetamol group, and 4.7% in the ibuprofen group ( Figure 1 ). There was no statistical difference between the groups (p=0.303). The results were similar between the paracetamol and the ibuprofen groups in terms of clinical and demographic characteristics, clinical outcomes, hepatic and renal function tests ( Table 2 , 3 and 4 ).

**Figure 1:** – Flowchart of the study population. PDA: patent ductus arteriosus.

**Table 2:** – Clinical and Demographical Characteristics of the Study Population

Clinical and demographical characteristics	Paracetamol (n: 101, 37.4%)	Ibuprofen (n: 169, 62.6%)	p-value
Gestational age, weeks,^a^	28 (2.8)	28 (2)	0.653
Birth weight, g,^a^	1042 (426)	1020 (290)	0.329
Male,^b^	53 (52.4)	79 (47.6)	0.381
1. min. Apgar,^a^	5 (2)	5 (2)	0.112
5. min. Apgar,^a^	7 (2)	8 (1)	0.153
Antenatal steroids,^b^	71 (70.2)	119 (68.6)	0.124
Duration of MV, days,^a^	3 (8)	2 (5)	0.270
Duration of NIV, days,^a^	12 (16)	9 (12)	0.980
Oxygen supplementation, days,^a^	42 (36)	30 (33)	0.388
Day of full enteral feeding, days,^a^	17 (11)	16 (8)	0.131
Length of stay in hospital, days,^a^	76 (45)	66 (30)	0.861

*MV: mechanical ventilation; NIV: non-invasive ventilation.^a^Median (interquartile range),^b^n (%).*

**Table 3:** – Clinical Outcomes of Study Groups

Clinical and demographical characteristics	Paracetamol (n: 101, 37.4%)	Ibuprofen (n: 169, 62.6%)	p-value
RDS,^a^	84 (83.1)	131 (77.5)	0.279
IVH, grade ≥3,^a^	12 (11.8)	13 (7.7)	0.279
NEC, stage ≥2,^a^	3 (2)	4 (2.3)	0.524
BPD,^a^	22 (21.7)	34 (20.1)	0.421
ROP,^a^	15 (14.8)	24 (14.2)	0.255
EOS,^a^	18 (17.8)	28 (16.5)	0.867
LOS,^a^	35 (34.6)	44 (26)	0.454
Gastrointestinal bleeding,^a^	-	4 (2.3)	0.321
Pulmonary hemorrhage,^a^	2 (2)	5 (2.9)	0.514
Mortality,^a^	17 (19.8)	19 (11.2)	0.131
2^nd^course treatment,^a^	26 (25.7)	46 (27.2)	0.312
3^rd^course treatment,^a^	13 (12.8)	23 (13.6)	0.191
PDA ligation,^a^	6 (5.9)	8 (4.7)	0.303

*^a^n (%). BPD: bronchopulmonary dysplasia; EOS: early neonatal sepsis; PDA: patent ductus arteriosus; IVH: intraventricular hemorrhage; LOS: late onset neonatal sepsis; NEC: necrotizing enterocolitis; RDS: respiratory distress syndrome; ROP: retinopathy of prematurity.*

**Table 4:** – Evaluation of hepatic and renal function tests after the first course of treatment

Laboratory values	Intravenous paracetamol		p-value	Intravenous ibuprofen		p-value	p-value*
	
	Pre-treatment	Post-treatment		Pre-treatment	Post-treatment		
BUN (mg/dL),^a^	40 (24)	44 (28)	0.352	42 (25)	42 (11-92)	0.926	0.667
Serum creatinine (mg/dL),^a^	0.8 (0.5)	0.8 (0.3)	0.339	0.7 (0.4)	0.8 (0.4)	0.116	0.452
Serum AST (Units/L),^a^	28 (10)	28 (17)	0.758	28 (13)	28 (15)	0.995	0.844
Serum ALT (Units/L),^a^	23 (12)	20 (13)	0.571	24 (14)	22 (12)	0.237	0.707

ALT: alanine aminotransferase; AST: aspartate amino transferase; BUN: blood urea nitrogen. *p-value for post-treatment measurements between the groups. a Median (interquartile range).

## Discussion

Our results have shown that IV paracetamol and IV ibuprofen are similarly effective when used as the first-line treatment option to close PDA. Moreover, both IV drugs were well tolerated for side effects on kidney and liver, and gastrointestinal and pulmonary complications. Additionally, there was no difference between the groups in terms of premature morbidity and mortality. Since most (90%) IVH and pulmonary hemorrhage cases occur before 72 hours of life, any treatment starting beyond that period should not be capable of reducing their incidence, therefore no difference between the drugs used in our study would be expected.^26^

Ductus arteriosus is a vital anatomical formation that connects pulmonary and systemic circulation in the fetus. The main factors that cause DA patency in intrauterine life are low oxygen pressure, PG and nitric oxide. Increased oxygen levels and decreased Prostaglandin E2 (PGE2) immediately after delivery allow functional ductal closure. Ductal patency disrupts both hemodynamics in premature infants and contributes to prematurity-related morbidity and mortality.^27^ Therefore, once an hsPDA is detected, two main factors (oxygen and PG levels) that provide vasodilation of the ductus should be manipulated to ensure ductal closure. Indomethacin, ibuprofen and paracetamol, which inhibit PG synthesis from arachidonic acid, thus providing vasoconstriction, are used for ductal closure. PG synthase is the main enzyme that converts arachidonic acid into PG. This enzyme has two catalytic activities, including COX (-1, -2, -3) and peroxidase. Indomethacin and ibuprofen inhibit COX-1 and -2 enzymes, and paracetamol inhibits the enzyme COX-3 and peroxidase, thereby inhibiting PG synthesis. While peroxidase, the target enzyme of paracetamol, can be activated at low peroxide levels, COX, the target enzyme of ibuprofen, is activated at higher peroxide levels. Therefore, paracetamol is more effective than ibuprofen in hypoxia.^2^

Considering the side effects of indomethacin and ibuprofen, new treatment methods were needed to reduce the need for ligation. Oral paracetamol was found to be effective in the treatment of PDA in 5 patients with PDA who did not respond to ibuprofen in the first case series reported by Hammerman et al.^28^ In the first studies, paracetamol was not used as the first-line drug, but as an alternative drug to cases where COX inhibitors were ineffective or contraindicated.^28^ Then, paracetamol was used as the first-line treatment to close the ductus arteriosus.^1 , 2 , 10^ Previous studies have been conducted to investigate the efficacy and safety of oral ibuprofen compared to oral paracetamol to clarify whether paracetamol can be used as a first-line treatment for ductal closure in preterm infants. Generally, in previous studies, the efficacy and reliability of oral forms of drugs used in hsPDA treatment have been evaluated.^1 , 29 - 34^

Our study, in addition to determining the efficacy of IV paracetamol in the treatment of hsPDA, aimed to compare it with IV ibuprofen. Based on our findings, whilst hsPDA closure rates were 74.3% with IV paracetamol, this ratio was 72.8% with IV ibuprofen, and there was no difference in both groups. In many studies, this rate was found to be approximately 70–85%, and was similar to the results of our study. Similar results were found in our study for side effects, complications and clinical outcomes as well.^2^ However, it was seen that the number of cases in the groups of previous studies ranged from 10 to 80.^1 , 2 , 10 , 11 , 35^ Therefore, our results might be more robust or stronger and reliable than other studies due to a large number of cases.

Although there is evidence to show that paracetamol is as effective as ibuprofen, there are studies that have found conflicting results. For instance, Lu et al.^12^ showed that paracetamol was less effective than ibuprofen for the closure of PDA in newborns, and this effect was reduced even more in very low birth weight (VLBW) or extremely low birth weight babies.^12^ Similarly, in a study by Sallmon, it was reported that in parallel with the 27.5% PDA closure rate observed after paracetamol treatment, the total closure rate of PDA was only 21.1% following the administration of paracetamol in VLBW infants.^36^ In addition, some studies have reported that paracetamol is not as effective as ibuprofen.^37 , 38^ Generally, paracetamol is used as an alternative option in selected patients, in which the ibuprofen could not be used, such as preterm infants with sepsis, whose general condition is poor, whose organ functions are not appropriate. Logically, the possible success of paracetamol is decreased for those patients.^15^ Therefore, as in our study, it would be more appropriate to evaluate that paracetamol can be used as the first choice based on the results of studies comparing the efficacy of paracetamol and ibuprofen in the treatment of hsPDA. Our study showed that IV paracetamol was similarly effective as IV ibuprofen in the treatment of hsPDA. Therefore, we suggest that IV paracetamol can be used as the first-line treatment for hsPDA as well as IV ibuprofen.

Previous studies have been conducted to compare the efficacy and safety of oral forms of the drugs for the treatment of hsPDA.^1 , 2 , 10 - 13^ Also, there are limited studies with IV forms. A study by Roofthooft et al.^14^ has reported that IV paracetamol treatment is not effective for PDA closure in VLBW babies after failure of IV ibuprofen treatment.^14^ In this study, paracetamol therapy was not recommended for PDA closure for infants >2 weeks of age after birth. However, paracetamol has been reported to be effective when used as a first-line treatment for PDA. Furthermore, Valerio et al.^25^ have found that IV paracetamol is effective in closing PDA for both “primary care” and “recovery” therapy.^25^ In another IV paracetamol and IV ibuprofen comparison study, it was stated that paracetamol could become the preferred treatment for the management of PDA, mainly due to its more favorable side effect profile.^9^ Our results also supported this information. Also, the side effects of ibuprofen are sometimes a disadvantage for its recommendation.^10 , 12^ In some studies, similar to our results, it is reported that there is no difference in terms of the side effect profile of both paracetamol and ibuprofen.^1 , 2 , 9 , 11^ In addition, supporting the previous studies, we found that surgical ligation rates were similarly decreased in infants with hsPDA treated either with paracetamol or ibuprofen.^1 , 2 , 11^

However, it is still being investigated which drug will be given in the safest and most effective way. Despite all these contradictory results, a recent Cochrane metanalysis by Ohlsson and Shah^16^ has stated that further studies are required before routine use of paracetamol for the first-line treatment of hsPDA.^16^ We think that the results of our study will shed light on this issue. According to our results, when IV paracetamol was given as the first-line treatment option for PDA closure, it was found to be as effective as IV ibuprofen without any side effects.

Our study had some limitations due to its retrospective nature. Other parameters such as hourly urine output, bilirubin level of patients in the treatment groups could not be evaluated. There is lack of data to recommend the first-line treatment of PDA in terms of morbidities, as there are other factors that should be considered. For example: the only drug that could reduce IVH in studies was early indomethacin. It would be more appropriate to show the efficacy of paracetamol in closing PDA and suggest randomized multicenter studies that could certify its safety and capacity of reducing negative clinical outcomes.

## Conclusions

When paracetamol was used as the first-line treatment option for the medical treatment of PDA, it was found to be similarly effective as ibuprofen. Additionally, there was no difference between two drugs in terms of premature morbidity and mortality. Multicenter randomized controlled studies on this subject will help to determine the first-line treatment of hsPDA.
